# Supplementary material for: Magnitude and predictors of anemia among adult solid cancer patients in Hawassa, Ethiopia: A cross-sectional study
Source: PLoS One. 2026 Jul 1;21(7):e0342280. doi: 10.1371/journal.pone.0342280 (PMC13322556; doi:10.1371/journal.pone.0342280)
Supplement: Attachment — Submitted filename: Reponse to academic editor and reviewers.docx [file pone.0342280.s002.docx]

**Dear Academic Editor,**

Thank you for allowing me to submit a revised version of the paper entitled “**Magnitude and Predictors of Anemia Among Adult Solid Cancer Patients in Hawassa, Ethiopia: A Cross-Sectional Study**.” I sincerely appreciate the time and effort you and the reviewers invested in providing insightful comments on the manuscript. These suggestions have greatly improved the clarity and rigor of the work.

I have incorporated changes to reflect most of the reviewers’ recommendations, and all modifications are highlighted in the revised manuscript. Below, I provide a detailed point‑by‑point response to each comment and concern raised.

We have revised the manuscript to fully comply with PLOS ONE formatting and style requirements, including file naming conventions. The Data Availability Statement has been updated to designate the Institutional Review Board (IRB) of Hawassa University College of Medicine and Health Sciences as the non‑author institutional contact point. In addition, the dataset generated and analyzed during this study will be deposited in the Zenodo repository upon acceptance to ensure open access in accordance with PLOS ONE’s data sharing policy.

**Reviewer Comments and Author's response:**

**Reviewer #1**

**I. General Comment**

- **Title**: Hyphenate Cross-sectional (this is standard in medical/epidemiological writing). Capitalization: In title case, short linking words like “of,” “in,” and “a” are usually lowercased, as you have them

**Response:** Thank you for this observation. The title has been corrected to “Magnitude and Predictors of Anemia Among Adult Solid Cancer Patients in Hawassa, Ethiopia: A Cross‑sectional Study” with proper hyphenation and capitalization.

- **Editorial issues**: In the method section, line numbers 92–93, added “is” after “which”. Removed the second “city” as redundant. Capitalized City and Regional State for a formal, proper-noun style.

**Response:** Thank you for these suggestions. We have corrected the phrasing by adding “is,” removed the redundant “city,” and capitalized “City” and “Regional State” to ensure proper-noun style.

- **Consistency in the use of abbreviations**. Eg, HUCSH vs HU-CSH.

**Response:** Thank you for pointing this out. We have standardized the abbreviation throughout the manuscript to “HUCSH” for consistency.

- **Citation:** for instance, no citation for the study area, line numbers 92-99

**Response:** Thank you for noting this. We have added an appropriate citation to support the description of the study area in the Methods section (lines 92–99).

**II. Specific comments to be addressed**
Method:

• The study appears to have been conducted five years ago and, therefore, may not accurately represent the current burden of anemia among cancer patients. How do the authors justify the relevance of these data? In addition, further clarification on how the sample size was allocated would be helpful, and it may be preferable to present this more specifically.

**Response:** We appreciate this important observation. The study was conducted between June 1 and October 30, 2021, and therefore does not reflect data collected five years ago. The findings remain relevant, as anemia continues to be a common comorbidity among cancer patients in our setting, and the study provides valuable baseline evidence for clinical practice and future research. Moreover, there is still limited or no updated research available in this area, which underscores the importance of our work. The total sample size of 405 was determined using Epi Info software with a 95% confidence level, 80% power, and an assumed anemia prevalence of 23%, including a 10% non‑response rate. According to patient flow, 205 participants were allocated to HUCSH, while 100 were assigned to Yanet Specialized Medical Center and 100 to Alatyon General Hospital. Eligible patients were systematically selected from oncology departments using cytology/histopathology records.

• With respect to ethical approval, it is unclear why the authors obtained verbal consent in addition to written informed consent. Could they clarify the rationale for this approach?

**Response**: Thank you for raising this point. We accept the comment and have corrected the wording. Written informed consent was obtained from all participants, as required by the Institutional Review Board. The mention of “verbal consent” in the earlier draft was not appropriate, and we have revised the manuscript to reflect that only written informed consent was obtained.

Result
• The authors state that study participants were included after cytological or histopathological confirmation. It would be more informative if they also presented the histopathological findings for patients with solid tumors and for controls.

**Response:** Thank you for this valuable suggestion. All study participants were included after cytological or histopathological confirmation of solid tumors. While detailed histopathological subtyping (e.g., ductal vs. lobular breast carcinoma) was not abstracted from medical records for this study, the distribution of cancer types and their respective anemia prevalence is already presented in Table 1. This provides the requested information on cancer type findings among anemic and non‑anemic patients. We believe this presentation adequately addresses the reviewer’s concern by showing how anemia prevalence varies across cancer categories, even though finer histopathological subtyping was not available in our dataset

• **Table 1 (Occupation):** The authors should consider reclassifying the occupational categories. What exactly does “unemployed” mean in this context? How were merchants, investors, company owners, or drivers classified? Are farmers considered unemployed? Clarification and more precise categorization are needed.

**Response:** Thank you for this important observation. In the original dataset, occupations were recorded in more detailed categories (government employee, private employee, merchant, daily laborer, farmer, housewife, non‑employed, student, and others). For analysis, these were reclassified into three broader groups: *Employed* (government and private employees, merchants, daily laborers, and others such as drivers or company owners), *Unemployed* (housewives, students, and those recorded as non‑employed), and *Farmers* (kept as a separate category due to their distinct socioeconomic profile in our setting). Farmers were not considered unemployed. We have clarified this classification in the revised manuscript to avoid ambiguity.

**• Figure 1:** The figure legend is unclear; for instance, the x-axis is not labeled. In addition, the figure title is in all uppercase and is stylistically inconsistent with the rest of the figures and text.

**Response:** Thank you for this observation. We initially revised Figure 1 to improve clarity by labeling the x‑axis, rewriting the legend, and reformatting the title to sentence case. However, since Reviewer #2 also found the figure redundant and suggested elimination, we have ultimately removed Figure 1 from the revised manuscript. The morphological distribution of anemia types is now presented clearly in the text, which avoids redundancy while ensuring clarity.

• **Recommendation part:**
Although most patients had normocytic anemia, which is consistent with cancer‑related anemia when confirmed, the authors recommend iron and vitamin B12 supplementation for these patients. This appears to go beyond the scope of the study, and the recommendation should be revised and better contextualized.

**Response:** Thank you for this valuable comment. We agree that our initial recommendation regarding iron and vitamin B12 supplementation extended beyond the scope of the present study. Our findings primarily demonstrated that most patients had normocytic anemia, which is consistent with cancer‑related anemia. We have therefore revised the recommendation to focus on the importance of routine anemia screening and appropriate management in cancer patients, while avoiding specific therapeutic suggestions that were not directly evaluated in this study.

**Reviewer #2**: The present study presents the results of original research regarding anemia in solid cancer patients in Ethiopia. On my own opinion, the paper seems to have a limited local interest and the data reported do not significantly differ from the those reported in other countries.

**Response:** Thank you for this observation. While we acknowledge that our findings on anemia patterns in solid cancer patients are consistent with reports from other countries, we believe that documenting these results in the Ethiopian context adds important value. Local data are essential for guiding clinical practice, informing national cancer care strategies, and contributing to global comparative research. Our study provides baseline evidence from Ethiopia, where such data have been limited, and thus addresses an important gap in the literature.

Specific observations:
1) **most cases (56,1%) have normocytic anemia**. This is not surprising: all patients suffer for a solid cancer and it is well known that the most frequent cause of normocytic anemia are chronic diseases. However, Authors stated that 16.5% of patients had a history of bleedings and about half of them were anemic. Considering that hemorrhages usually cause microcytic anemia, I would expect that they have a microcytic anemia. The Authors have to better describe this topic and explain their findings.

**Response:** Thank you for this important observation. We agree that hemorrhage typically results in microcytic anemia. In our study, however, most patients with a history of bleeding still presented with normocytic anemia. This finding likely reflects the multifactorial nature of anemia in cancer patients, where chronic disease and inflammation are dominant contributors even in those with bleeding episodes. It is also possible that the bleeding events were intermittent or of limited severity, and thus insufficient to produce sustained microcytosis. We have clarified this point in the Discussion section to better contextualize our findings.

2. Even if macrocytic anemia were uncommon, can the Authors find a correlation with decreased BMI?

**Response:** Thank you for this important observation. In our cohort, only one patient (0.9%) had macrocytic anemia (MCV > 100 fl). Because of this rarity, statistical correlation analysis with BMI could not be performed. We have clarified this explicitly in the Results section by reporting the frequency of macrocytic anemia and noting that no meaningful correlation with BMI was possible. Nonetheless, anemia overall was significantly associated with BMI category (χ² = 9.48, p = 0.002), and underweight patients had approximately twice the odds of being anemic compared to normal/overweight patients (AOR = 2.14, 95% CI: 1.28–3.55, p = 0.003). We have also highlighted in the Discussion that although macrocytic anemia was too uncommon to analyze, the broader relationship between low BMI and anemia was statistically significant.

3. Which other therapies, in addition to blood transfusions, were used in anemic patients?

**Response:** Thank you for this comment. In our study, among patients with a treatment plan for anemia, 62.2% received blood transfusions and 37.8% were prescribed iron supplementation. Other therapies such as folate or vitamin B12 supplementation were occasionally used in clinical practice but were not systematically recorded in our dataset.

4. I found the figure redundant and I think it can be eliminated.

**Response:** Thank you for this suggestion. We agree that the figure was redundant, as the morphological distribution of anemia types is already clearly presented in the text. We have therefore removed the figure from the revised manuscript to streamline presentation.

I look forward to hearing from you in due time regarding my submission and to respond to any further questions and comments you may have.

Sincerely,

Abebe Melis Nisro

Corresponding author

May, 2026
