# Supplementary material for: Magnitude and predictors of anemia among adult solid cancer patients in Hawassa, Ethiopia: A cross-sectional study
Source: PLoS One. 2026 Jul 1;21(7):e0342280. doi: 10.1371/journal.pone.0342280 (PMC13322556; doi:10.1371/journal.pone.0342280)
Supplement: Peer review history

# Peer review: Decision Letter 0

**DOI:** 10.1371/journal.pone.0342280.r001

**Authors:** Berhanu Feleke

30 Mar 2026

PONE-D-26-02820

Magnitude and Predictors of Anemia Among Adult Solid Cancer Patients in Hawassa, Ethiopia: A Cross-Sectional Study.

PLOS One

Dear Dr. Nisro,

Thank you for submitting your manuscript to PLOS ONE. After careful consideration, we feel that it has merit but does not fully meet PLOS ONE’s publication criteria as it currently stands. Therefore, we invite you to submit a revised version of the manuscript that addresses the points raised during the review process.

Please submit your revised manuscript by May 14 2026 11:59PM. If you will need more time than this to complete your revisions, please reply to this message or contact the journal office at plosone@plos.org. When you're ready to submit your revision, log on to https://www.editorialmanager.com/pone/ and select the 'Submissions Needing Revision' folder to locate your manuscript file.

Please include the following items when submitting your revised manuscript:

- A letter that responds to each point raised by the academic editor and reviewer(s). You should upload this letter as a separate file labeled 'Response to Reviewers'.
- A marked-up copy of your manuscript that highlights changes made to the original version. You should upload this as a separate file labeled 'Revised Manuscript with Track Changes'.
- An unmarked version of your revised paper without tracked changes. You should upload this as a separate file labeled 'Manuscript'.

If you would like to make changes to your financial disclosure, please include your updated statement in your cover letter. Guidelines for resubmitting your figure files are available below the reviewer comments at the end of this letter.

If applicable, we recommend that you deposit your laboratory protocols in protocols.io to enhance the reproducibility of your results. Protocols.io assigns your protocol its own identifier (DOI) so that it can be cited independently in the future. For instructions see: https://journals.plos.org/plosone/s/submission-guidelines#loc-laboratory-protocols. Additionally, PLOS ONE offers an option for publishing peer-reviewed Lab Protocol articles, which describe protocols hosted on protocols.io. Read more information on sharing protocols at https://plos.org/protocols?utm_medium=editorial-email&utm_source=authorletters&utm_campaign=protocols.

As the corresponding author, your ORCID iD is verified in the submission system and will appear in the published article. PLOS supports the use of ORCID, and we encourage all coauthors to register for an ORCID iD and use it as well. Please encourage your coauthors to verify their ORCID iD within the submission system before final acceptance, as unverified ORCID iDs will not appear in the published article. *Only*  the individual author can complete the verification step; PLOS staff *cannot*  verify ORCID iDs on behalf of authors.

We look forward to receiving your revised manuscript.

Kind regards,

Berhanu Elfu Feleke, Ph.D.

Academic Editor

PLOS One

Journal Requirements:

When submitting your revision, we need you to address these additional requirements.

1.Please ensure that your manuscript meets PLOS ONE's style requirements, including those for file naming. The PLOS ONE style templates can be found at

https://journals.plos.org/plosone/s/file?id=wjVg/PLOSOne_formatting_sample_main_body.pdf and

https://journals.plos.org/plosone/s/file?id=ba62/PLOSOne_formatting_sample_title_authors_affiliations.pdf

2. In the online submission form you indicate that your data is not available for proprietary reasons and have provided a contact point for accessing this data. Please note that your current contact point is a co-author on this manuscript. According to our Data Policy, the contact point must not be an author on the manuscript and must be an institutional contact, ideally not an individual. Please revise your data statement to a non-author institutional point of contact, such as a data access or ethics committee, and send this to us via return email. Please also include contact information for the third party organization, and please include the full citation of where the data can be found.

3. When completing the data availability statement of the submission form, you indicated that you will make your data available on acceptance. We strongly recommend all authors decide on a data sharing plan before acceptance, as the process can be lengthy and hold up publication timelines. Please note that, though access restrictions are acceptable now, your entire data will need to be made freely accessible if your manuscript is accepted for publication. This policy applies to all data except where public deposition would breach compliance with the protocol approved by your research ethics board. If you are unable to adhere to our open data policy, please kindly revise your statement to explain your reasoning and we will seek the editor's input on an exemption. Please be assured that, once you have provided your new statement, the assessment of your exemption will not hold up the peer review process.

4. If the reviewer comments include a recommendation to cite specific previously published works, please review and evaluate these publications to determine whether they are relevant and should be cited. There is no requirement to cite these works unless the editor has indicated otherwise.

[Note: HTML markup is below. Please do not edit.]

Reviewer's Responses to Questions

**Comments to the Author**

1. Is the manuscript technically sound, and do the data support the conclusions?

The manuscript must describe a technically sound piece of scientific research with data that supports the conclusions. Experiments must have been conducted rigorously, with appropriate controls, replication, and sample sizes. The conclusions must be drawn appropriately based on the data presented.

Reviewer #1: Yes

Reviewer #2: Partly

**********

2. Has the statistical analysis been performed appropriately and rigorously?

Reviewer #1: No

Reviewer #2: Yes

**********

3. Have the authors made all data underlying the findings in their manuscript fully available?

The PLOS Data policy requires authors to make all data underlying the findings described in their manuscript fully available without restriction, with rare exception (please refer to the Data Availability Statement in the manuscript PDF file). The data should be provided as part of the manuscript or its supporting information, or deposited to a public repository. For example, in addition to summary statistics, the data points behind means, medians and variance measures should be available. If there are restrictions on publicly sharing data—e.g. participant privacy or use of data from a third party—those must be specified.

Reviewer #1: No

Reviewer #2: Yes

**********

4. Is the manuscript presented in an intelligible fashion and written in standard English?

PLOS ONE does not copyedit accepted manuscripts, so the language in submitted articles must be clear, correct, and unambiguous. Any typographical or grammatical errors should be corrected at revision, so please note any specific errors here.

Reviewer #1: Yes

Reviewer #2: Yes

**********

5. Review Comments to the Author

Please use the space provided to explain your answers to the questions above. You may also include additional comments for the author, including concerns about dual publication, research ethics, or publication ethics. (Please upload your review as an attachment if it exceeds 20,000 characters)

Reviewer #1: Anemia is a common problem in developing countries, especially in patients with cancer and cardiac disorders. In this study, the authors explored the magnitude of anemia and aimed to identify predictors for the condition. They investigated the burden of anemia using clinical samples and questionnaires and presented their findings. However, there remain general and specific issues that must be addressed.

I. General comments

The following are some of the general comments. For instance:

• Title: Hyphenate Cross-sectional (this is standard in medical/epidemiological writing). Capitalization: In title case, short linking words like “of,” “in,” and “a” are usually lowercased, as you have them.

• Editorial issues: In the method section, line numbers 92–93, added “is” after “which”. Removed the second “city” as redundant. Capitalized City and Regional State for a formal, proper-noun style.

• Consistency in the use of abbreviations. Eg, HUCSH vs HU-CSH.

• Citation: for instance, no citation for the study area, line numbers 92-99

II. Specific comments to be addressed

Method:

• The study appears to have been conducted five years ago and, therefore, may not accurately represent the current burden of anemia among cancer patients. How do the authors justify the relevance of these data? In addition, further clarification on how the sample size was allocated would be helpful, and it may be preferable to present this more specifically.

• With respect to ethical approval, it is unclear why the authors obtained verbal consent in addition to written informed consent. Could they clarify the rationale for this approach?

Result

• The authors state that study participants were included after cytological or histopathological confirmation. It would be more informative if they also presented the histopathological findings for patients with solid tumors and for controls.

• Table 1 (Occupation): The authors should consider reclassifying the occupational categories. What exactly does “unemployed” mean in this context? How were merchants, investors, company owners, or drivers classified? Are farmers considered unemployed? Clarification and more precise categorization are needed.

• Figure 1: The figure legend is unclear; for instance, the x-axis is not labeled. In addition, the figure title is in all uppercase and is stylistically inconsistent with the rest of the figures and text.

Recommendation part:

Although most patients had normocytic anemia, which is consistent with cancer‑related anemia when confirmed, the authors recommend iron and vitamin B12 supplementation for these patients. This appears to go beyond the scope of the study, and the recommendation should be revised and better contextualized.

My decision:

- Accept pending major revisions, contingent upon addressing the points outlined above.

Reviewer #2: The present study presents the results of an original research regarding anemia in solid cancer patients in Ethiopia. On my own opinion, the paper seems to have a limited local interest and the data reported do not significantly differe from the those reported in other countries.

Specific observations:

1) most cases (56,1%) have normocytic anemia. This is not surprising: all patients suffer for a solid cancer and it is well known that the most frequent cause of normocytic anemia are chronic diseases. However, Authors stated that 16.5% of patients had a history of bleedings and about half of them were anemic. Considering that hemorrhages usually cause microcytic anemia, I would expect that they have a microcytic anemia. The Authors have to better describe this topic and explain their findings

2) Even if macrocytic anemia were uncommon, can the Authors find a correlation with decreased BMI?

3) Which other therapies, in addition to blood transfusions, were used in anemic patients?

4) I found the figure redundant and I think it can be eliminated.

I think that the data may be better summarized in a

**********

6. PLOS authors have the option to publish the peer review history of their article (what does this mean?). If published, this will include your full peer review and any attached files.

If you choose “no”, your identity will remain anonymous but your review may still be made public.

**Do you want your identity to be public for this peer review?**  For information about this choice, including consent withdrawal, please see our Privacy Policy.

Reviewer #1: No

Reviewer #2: No

**********

[NOTE: If reviewer comments were submitted as an attachment file, they will be attached to this email and accessible via the submission site. Please log into your account, locate the manuscript record, and check for the action link "View Attachments". If this link does not appear, there are no attachment files.]

To ensure your figures meet our technical requirements, please review our figure guidelines: https://journals.plos.org/plosone/s/figures

You may also use PLOS’s free figure tool, NAAS, to help you prepare publication quality figures: https://journals.plos.org/plosone/s/figures#loc-tools-for-figure-preparation.

NAAS will assess whether your figures meet our technical requirements by comparing each figure against our figure specifications.

# Author comment: Author response to Decision Letter 1

**DOI:** 10.1371/journal.pone.0342280.r002

12 May 2026

We have carefully revised the manuscript in line with the reviewers’ and editor’s comments. The title, formatting, and editorial issues have been corrected, and clarifications have been added to the Methods, Results, and Discussion sections. The Data Availability Statement has been updated to comply with PLOS ONE’s policy, with the dataset already deposited in Zenodo and the Institutional Review Board of Hawassa University College of Medicine and Health Sciences designated as the non‑author contact point. Figures and recommendations have been streamlined, and all reviewer concerns have been addressed in detail. We believe the revised manuscript now fully meets the journal’s requirements.

Attachment Submitted filename: Reponse to academic editor and reviewers.docx

# Editor report: Decision Letter 1

**DOI:** 10.1371/journal.pone.0342280.r003

**Authors:** Berhanu Feleke, Berhanu Feleke

15 Jun 2026

Magnitude and Predictors of Anemia Among Adult Solid Cancer Patients in Hawassa, Ethiopia: A Cross-Sectional Study.

PONE-D-26-02820R1

Dear Dr. Nisro,

We’re pleased to inform you that your manuscript has been judged scientifically suitable for publication and will be formally accepted for publication once it meets all outstanding technical requirements.

Within one week, you’ll receive an e-mail detailing the required amendments. When these have been addressed, you’ll receive a formal acceptance letter and your manuscript will be scheduled for publication.

An invoice will be generated when your article is formally accepted. Please note, if your institution has a publishing partnership with PLOS and your article meets the relevant criteria, all or part of your publication costs will be covered. Please make sure your user information is up-to-date by logging into Editorial Manager at Editorial Manager® and clicking the ‘Update My Information' link at the top of the page. For questions related to billing, please contact billing support.

If your institution or institutions have a press office, please notify them about your upcoming paper to help maximize its impact. If they’ll be preparing press materials, please inform our press team as soon as possible -- no later than 48 hours after receiving the formal acceptance. Your manuscript will remain under strict press embargo until 2 pm Eastern Time on the date of publication. For more information, please contact onepress@plos.org.

Kind regards,

Berhanu Elfu Feleke, Ph.D.

Academic Editor

PLOS One

# Editor report: Acceptance letter

**DOI:** 10.1371/journal.pone.0342280.r004

**Authors:** Berhanu Feleke, Berhanu Feleke

PONE-D-26-02820R1

PLOS One

Dear Dr. Nisro,

I'm pleased to inform you that your manuscript has been deemed suitable for publication in PLOS One. Congratulations! Your manuscript is now being handed over to our production team.

At this stage, our production department will prepare your paper for publication. This includes ensuring the following:

* All references, tables, and figures are properly cited

* All relevant supporting information is included in the manuscript submission,

* There are no issues that prevent the paper from being properly typeset

You will receive further instructions from the production team, including instructions on how to review your proof when it is ready. Please keep in mind that we are working through a large volume of accepted articles, so please give us a few days to review your paper and let you know the next and final steps.

Lastly, if your institution or institutions have a press office, please let them know about your upcoming paper now to help maximize its impact. If they'll be preparing press materials, please inform our press team within the next 48 hours. Your manuscript will remain under strict press embargo until 2 pm Eastern Time on the date of publication. For more information, please contact onepress@plos.org.

You will receive an invoice from PLOS for your publication fee after your manuscript has reached the completed accept phase. If you receive an email requesting payment before acceptance or for any other service, this may be a phishing scheme. Learn how to identify phishing emails and protect your accounts at https://explore.plos.org/phishing.

If we can help with anything else, please email us at customercare@plos.org.

Thank you for submitting your work to PLOS ONE and supporting open access.

Kind regards,

PLOS ONE Editorial Office Staff

on behalf of

Dr Berhanu Elfu Feleke

Academic Editor

PLOS One
